# The Role of Wearable Technology in Measuring and Supporting Patient Outcomes Following Total Joint Replacement: Review of the Literature

**DOI:** 10.2196/39396

**Published:** 2023-01-12

**Authors:** Gregory Iovanel, David Ayers, Hua Zheng

**Affiliations:** 1 Department of Orthopedics and Physical Rehabilitation UMass Chan Medical School Worcester, MA United States

**Keywords:** total joint replacement, wearables, osteoarthritis, rehabilitation, mobility

## Abstract

**Background:**

The incidence rate of total joint replacement (TJR) continues to increase due to the aging population and the surgery that is very successful in providing pain relief to and improving function among patients with advanced knee or hip arthritis. Improving patient outcomes and patient satisfaction after TJR remain important goals. Wearable technologies provide a novel way to capture patient function and activity data and supplement clinical measures and patient-reported outcome measures in order to better understand patient outcomes after TJR.

**Objective:**

We examined the current literature to evaluate the potential role of wearable devices and compare them with existing methods for monitoring and improving patient rehabilitation and outcomes following TJR.

**Methods:**

We performed a literature search by using the research databases supported by the University of Massachusetts Chan Medical School’s Lamar Soutter Library, including PubMed and Scopus, supplemented with the Google Scholar search engine. A specific search strategy was used to identify articles discussing the use of wearable devices in measuring and affecting postoperative outcomes of patients who have undergone TJR. Selected papers were organized into a spreadsheet and categorized for our qualitative literature review to assess how wearable data correlated with clinical measures and patient-reported outcome measures.

**Results:**

A total of 9 papers were selected. The literature showed the impact of wearable devices on evaluating and improving postoperative functional outcomes. Wearable-collected data could be used to predict postoperative clinical measures, such as range of motion and Timed Up and Go times. When predicting patient-reported outcomes, specifically Hip Disability and Osteoarthritis Outcome Scores/Knee Injury and Osteoarthritis Outcome Scores and Veterans RAND 12-Item Health Survey scores, strong associations were found between changes in sensor-collected data and changes in patient-reported outcomes over time. Further, the step counts of patients who received feedback from a wearable improved over time when compared to those of patients who did not receive feedback.

**Conclusions:**

These findings suggest that wearable technology has the potential to remotely measure and improve postoperative orthopedic patient outcomes. We anticipate that this review will facilitate further investigation into whether wearable devices are viable tools for guiding the clinical management of TJR rehabilitation.

## Introduction

Total joint replacement (TJR) has proven to be highly effective in relieving joint pain and improving physical function for millions of patients with advanced knee or hip osteoarthritis and continues to be one of the most commonly performed surgical procedures in the United States [1-3]. As this trend persists, increased attention must be paid toward effectively monitoring and coaching patients following surgery to ensure successful rehabilitation. Traditional assessments of postoperative recovery, such as the Timed Up and Go (TUG) and 6-minute walk tests, are considered gold standards for measuring mobility, balance, and walking ability [4]. However, these assessments require in-person monitoring by health care providers and do not replicate activities of daily living. Patient-reported outcomes (PROs) have been widely used to evaluate joint pain and physical function through standardized patient questionnaires. Patients report on how they perceive their health status without the interpretation of a medical professional. Although the assessment of PROs has become part of the standard of care in many orthopedic practices, the implementation of PRO capture, the maintenance of data integrity, data interpretation, and cost management are still challenging for many practices [5-9]. The internet-based remote monitoring of patient mobility data is an alternative method of collecting patient data following surgery that has recently been introduced and warrants further evaluation.

Wearable technologies provide a novel way to capture patient function and activity data and supplement clinical measures and PRO measures (PROMs) to better understand patient recovery after TJR. Wearable technologies, in the context of health care, refer to devices that can record real-time data from an individual while worn. These devices include accelerometers, which capture the acceleration of a limb or the entire body; gyroscopes, which measure orientation and angular velocity; and inertial measurement units—a more sophisticated technology that combines an accelerometer, gyroscope, and magnetometer and is capable of reporting the movement, orientation, and position in space of a person or object [10]. Many companies manufacture such devices that can be synced to a smartphone, computer, or tablet to transmit patient mobility data securely and instantly to health care providers via an internet-based application. Medical professionals are then able to track patients’ progress in real time and tailor rehabilitation regimens for patients to follow, based on the data obtained [11,12]. Such wearable technologies could offer the possibility of capturing real-time function data on the rehabilitation and recovery of patients who have undergone TJR and eliminating the need for direct supervision. In addition, a connected mobile app can be developed to collect PROMs, thereby minimizing the need for additional PROM capture tools [13]. Current research has shown the feasibility of wearable devices and their capability for motion and activity tracking [14]. However, it is not clear whether the activity data collected by wearable devices can serve as outcome measures or as adjuncts to support outcome monitoring. There is a dearth of consensus on whether wearables can be used as effective tools, can be aligned with standard clinical measures and PROMs, or can even improve outcomes.

To promote wearable use as part of rehabilitation programs following TJR, their impact on postoperative patient outcomes, as well as their accuracy in measuring these outcomes, must be further investigated. This paper seeks to review the current landscape of orthopedic wearables literature and assess the effectiveness of available devices with respect to evaluating and improving postoperative outcomes.

## Methods

A literature search was conducted by using the research databases supported by the University of Massachusetts Chan Medical School’s Lamar Soutter Library, including PubMed and Scopus, supplemented with the Google Scholar search engine. Articles published in English from 2004 to 2021 were reviewed. The search terms used to identify these articles are defined in Textbox 1.

**Literature search strategy (search terms used in the literature search strategy).**

**Term groupings and search terms**

- Wearable devices
*(“wearable”) AND (“devices” OR “technology”)*
- Patient
*(“total joint replacement” OR “total knee replacement” OR “total hip replacement”) AND “outcomes”*
- Rehabilitation
*“rehabilitation” OR “recovery”*

The inclusion criteria included English-language articles, research studies, and studies with wearable technology that focused on comparing wearable-collected data with clinical measures or PROMs or affecting patient outcomes. The exclusion criteria were articles focusing on wearable device design, study protocols, theoretical articles, books, or book chapters. Titles and abstracts of identified articles were screened to determine eligibility based on the inclusion and exclusion criteria. Since only a limited number of papers met the inclusion criteria, a full reading was conducted for all of the eligible papers.

The information was tabulated via a standardized Excel (Microsoft Corporation) form that was developed for this review, which included the first author’s name, year of publication, name and type of the wearable device, location where the device was worn, number of patients in the study, outcome measures, and study findings (Table 1). A narrative literature review of the selected articles was conducted by 2 reviewers, providing a qualitative overview of outcome measures, data collection methods, and main findings.

**Table 1:** Classification of selected articles (papers were organized by the first author’s name, year, wearable device, device type, device location, number of patients, outcome measures, and findings).

Authors, year	Wearable device	Device type	Device location	Patients, n	Outcome measure	Findings
Kwasnicki et al [15], 2015	e-AR (Imperial College London)	Accelerometer	Ear	14	TUG^a^ time and ROM^b^	The classification of patients into preoperative, normal, and 24-week postoperative groups based on outcomes was 89% accurate, while classification for all time intervals was 69% accurate.
Chiang et al [16], 2017	APDM OPAL (APDM Wearable Technologies)	Accelerometer, gyroscope, magnetometer, and barometer	Thigh and calf (2 sensors)	18	Satisfaction	Only 17% of patients felt uncomfortable with the sensor belt.
Bendich et al [17], 2019	Fitbit Flex (Fitbit LLC)	Accelerometer	Wrist	22	Daily step count, daily minutes active, HOOS/KOOS^c^, and VR-12^d^ score	Changes from preoperative levels to 6-week postoperative levels in “daily step count” and “daily minutes active” (collected with a wearable sensor) were strongly associated with improvements in HOOSs/KOOSs and VR-12 physical component scores (collected over the same period).
Chen et al [18], 2015	APDM OPAL	Accelerometer, gyroscope, and magnetometer	Chest, thigh, and calf (3 sensors)	10	ROM	The device was able to identify proper exercise posture 88.26% of the time.
Battenberg et al [19], 2017	Fitbit One (Fitbit LLC), Omron HJ-321 (Omron Corporation), Sportline 340 Strider (Sportline Inc), Fitbit Force (Fitbit LLC), Nike+ Fuelband SE (Nike Inc), and StepWatch Activity Monitor (Orthocare Innovations)	Fitbit One (accelerometer), Omron HJ-321 (pedometer and accelerometer), Sportline 340 Strider (pedometer), Fitbit Force (accelerometer), Nike+ Fuelband SE (accelerometer), and StepWatch Activity Monitor (accelerometer)	Fitbit One (waist), Omron HJ-321 (waist), Sportline 340 Strider (waist), Fitbit Force (wrist), Nike+ Fuelband SE (wrist), and StepWatch Activity Monitor (ankle)	30	Step count	The waist-based devices—Fitbit One and Omron HJ-321—were >90% accurate in counting steps for all activities, the wristband devices were <90% accurate for most activities, and the StepWatch Activity Monitor (ankle) was >95% accurate for lower cadence activities but undercounted running by 25%.
Toogood et al [20], 2016	Fitbit (Fitbit LLC)	Accelerometer	Ankle	33	Compliance	The mean compliance over 30 days was 26.7 days (89%).
Saporito et al [21], 2019	Custom	Accelerometer and barometer	Neck (pendant)	15	TUG time	A strong correlation (ρ=0.70) was observed between remote TUG times and standardized TUG times.
Van der Walt et al [22], 2018	Garmin Vivofit 2 (Garmin Ltd)	Accelerometer	Wrist	163	Step count	Participants receiving feedback on step goals from the device had significantly higher (*P*<.03) mean daily step counts than those of participants who did not receive any feedback from the device.
Kuiken et al [23], 2004	Custom	Goniometer	Knee	11	ROM and mean activity rate	After total knee arthroplasty, patients wearing a device providing feedback had higher mean total activity rates—a measure of ROM—on days when they did not receive feedback from the device (mean 22.5, SD 11.1 activity counts per hour) than on days when they did receive feedback (mean 15.1, SD 10.9 activity counts per hour), but this was not statistically significant (*P*=.11).

^a^TUG: Timed Up and Go.

^b^ROM: range of motion.

^c^HOOS/KOOS: Hip Disability and Osteoarthritis Outcome Score/Knee Injury and Osteoarthritis Outcome Score.

^d^VR-12: Veterans RAND 12-Item Health Survey.

The standard postoperative TJR outcome measures in this literature review included (1) assessments typically conducted in clinical settings, such as range of motion (ROM) assessments and the TUG test, and (2) PROMs, such as joint-specific outcome measures (Hip Disability and Osteoarthritis Outcome Score/Knee Injury and Osteoarthritis Outcome Score [HOOS/KOOS]), global health measures (Veterans RAND 12-Item Health Survey [VR-12]), patient satisfaction, and activity adherence.

## Results

A total of 9 articles that met the inclusion criteria were identified. The articles evaluated the mobility and activity data collected through the wearable devices and compared them with standard clinical outcome measures and PROMs.

### Correlation of Wearables and Clinical Measures

In evaluating ROM and TUG time, the wearables varied in accuracy. Kwasnicki et al [15] observed 14 patients who underwent total knee replacement and wore the e-AR accelerometer (Imperial College London) on the ear to conduct home-based mobility assessments. The authors compared a generated sensor score, which was based on sensor data, with the results of other assessment techniques (TUG test and knee ROM). They calculated Spearman ρ correlation coefficients between sensor scores and TUG and ROM measurements to assess the strength of association. They found that perioperative sensor scores correlated, albeit not significantly for all activities, with TUG time and ROM improvements. In another study that focused on TUG measurements, Saporito et al [21] collected standardized TUG data from 239 community-living older adults in a laboratory and sensor-based data on participants’ activities of daily living through a wearable pendant device for at least 3 days and developed a regularized linear model for estimating remote TUG times. Based on the device data of 15 patients who underwent total hip replacement, a strong correlation was observed between estimated remote TUG times and standardized TUG times via leave-one-out cross-validation.

### Correlation of Wearables and PROMs

Data from wearable devices may correlate with PROMs. Bendich et al [17] aimed to determine whether sensor-collected data could be used as predictors of PROMs. In their study, 22 patients who underwent TJR wore a Fitbit Flex (Fitbit LLC) device on the wrist, which allowed for the observation of potential associations between “daily step count” and “daily minutes active” data collected by the wearable and PROMs, specifically the HOOS/KOOS and VR-12, over time. The researchers found that changes observed in “daily step count” from before the operation to postoperative week 6 were strongly associated with changes in VR-12 scores, while changes observed in “daily minutes active” from before the operation to postoperative week 6 were strongly associated with changes in HOOSs/KOOSs.

### Impact of the Use of Wearables on Patient Outcomes

The authors of 2 articles discussed the impact of the use of wearable devices on postoperative TJR patient outcomes. Specifically, the researchers investigated how the ability of devices to offer feedback on exercise and rehabilitation to patients may impact patient outcomes. Van der Walt et al [22] randomized 163 patients who underwent TJR into 2 groups; one received feedback for their rehabilitation via the Garmin Vivofit 2 (Garmin Ltd) accelerometer, and the other did not receive any feedback. They found that the mean daily step counts of the group that received feedback were significantly higher than those of the group that did not receive feedback (43% higher in postoperative week 1, 33% higher in postoperative week 2, 21% higher in postoperative week 6, and 17% higher at postoperative month 6). Surprisingly, in a study with 11 patients who underwent total knee arthroplasty, Kuiken et al [23] found that patients who wore a device that provided feedback had a slightly higher mean total activity rate on days when they did not receive feedback from the device compared to that on days when they did receive feedback from the device, although this difference was not statistically significant.

Patients reported high satisfaction with and adherence for the use of wearable devices. A study by Chiang et al [16] found that in a group of 18 patients who underwent total knee replacement and wore a thigh- and calf-worn wearable, 83% reported no discomfort when wearing the device. In a study by Toogood et al [20] on device adherence, the mean compliance rate for wearing an ankle-based Fitbit accelerometer (Fitbit LLC) among 33 patients who underwent total hip replacement was 89% (26.7/30 days). Although this study noted that devices were worn for 24 hours per day, apart from during washing, the daily duration of use was not specifically mentioned in the other selected studies.

### Device Data Accuracy Evaluation

Several devices were found to be generally accurate in counting steps. Battenberg et al [19] tested the accuracy of several widely used wearable devices in a convenience sample of 30 healthy participants. They found that the waist-worn Fitbit One (Fitbit LLC) and Omron HJ-321 (Omron Corporation) had greater than 90% accuracy in step counting during all activities; the wristband devices, such as the Fitbit Force (Fitbit LLC) and Nike+ Fuelband SE (Nike Inc), had less than 90% accuracy for most activities; and the ankle-worn StepWatch Activity Monitor (Orthocare Innovations) was greater than 95% accurate when counting steps during lower cadence activities but undercounted steps during running by 25%. In a study by Chen at al [18], 10 healthy participants, while wearing 3 APDM OPAL (APDM Wearable Technologies) sensors on the chest, thigh, and calf, performed 3 different rehabilitation exercises that were designed for patients with knee osteoarthritis to manage rehabilitation progress at home. The device was found to have an overall recognition accuracy of 97% for exercise type classification and an overall recognition accuracy of 88% for proper exercise posture.

## Discussion

### Wearable Data Can Be Used as Alternative Outcome Measures

Postoperative TJR recovery remains a black box to health care providers until patients report to a clinic or respond to a survey. With adequate implementation and the ability to collect data continuously, even from a remote setting, wearable devices can help health care providers to monitor progress consistently and detect early problems in rehabilitation [24]. The literature shows that function and activity data obtained from wearables, including step count and exercise tracking data, correlate with both clinical outcomes and PROMs [15,17,21]. Such wearable data are able to provide measures of patients’ objective functional outcomes that are comparable with standard clinical metrics and patient surveys. In addition, the opportunity to regularly monitor patients in real time and allow for direct feedback from and communication with health care providers can alleviate the inconveniences of unnecessary office visits and costs; patients with good progress can continue at-home rehabilitation, while patients with poor progress can be alerted to proactively visit a clinic before permanent complications occur. Further research is however needed to evaluate device bias and data accuracy to make sure that wearable results are reliable.

There has also been some support in the literature for the use of monitoring insoles, particularly for the purpose of load and gait analysis. Although preliminary findings suggest that monitoring insoles have good accuracy in measuring foot load distribution and natural gait, the few studies that have been performed are limited by small sample sizes [25,26]. Additional investigations with larger data sets will be needed.

### Wearables Can Be Used to Improve Outcomes

In addition to generating data that correlate with established outcomes, wearables can also be used to improve outcomes overall by more actively engaging patients in exercise and activity [24,27]. Indeed, devices connected to mobile apps can provide feedback to patients regarding their rehabilitation routines, and the mobility metrics, such as daily step count, of patients who received such feedback significantly improved when compared to those of patients who did not receive feedback [22]. Additionally, the ability of these wearables to provide daily exercise reminders to patients and plot their progress over time sustained patients’ motivation and further contributed to outcome improvement [28,29].

### Wearables and Apps Can Be Included in Future Health IT Infrastructure

As orthopedic clinical research has progressed, more data sources have emerged from which to monitor and guide patient rehabilitation and care following TJR. Whereas most patient data previously originated from electronic health records, direct patient-generated data in the form of PROMs or outcomes tracked and collected by wearables aptly supplement clinically collected data. Particularly, the ability of wearables to generate objective, continuous data showing trends in patient progress is unique in comparison to PROMs, which provide subjective data from predetermined time points, and electronic health record data, which are only collected during patients’ point-of-care visits and require medical professionals’ involvement. Moreover, with the increased emphasis on telemedicine, particularly since the COVID-19 pandemic, the remote monitoring of patient recovery via wearables represents a potential new path toward collecting patient data and guiding clinical decision-making [30,31]. These novel applications emphasize the role of wearables in the future of health IT infrastructure.

### Challenges

There are still challenges to the implementation of wearable technology. Technical support will be needed for device calibration and data collection. Some research teams have assisted in the use of wearables during appointments scheduled at patients’ homes [15], hospital wards, or outpatient clinics [16]. Patients also need to be provided with training and guidance before and during the study period to ensure proper device mounting and use. Additionally, standardization must be established across different devices and across data collection in different settings to ensure that data are comparable and meaningful.

### Conclusion

This review discusses the current state of the literature regarding the effectiveness of wearable devices in measuring and improving TJR outcomes, as well as the future directions of wearable device use. Wearable technologies have great potential for assessing and enhancing patients’ postoperative physical function. Wearables can be effective, alternative tools for evaluating TJR outcomes, as early findings have shown correlations among wearable-recorded data, PROMs, and clinical outcomes. The implementation and standardization of wearables should be addressed in future research.

## Abbreviations

HOOS/KOOS: Hip Disability and Osteoarthritis Outcome Score/Knee Injury and Osteoarthritis Outcome Score
PRO: patient-reported outcome
PROM: patient-reported outcome measure
ROM: range of motion
TJR: total joint replacement
TUG: Timed Up and Go
VR-12: Veterans RAND 12-Item Health Survey
